# Repetitive negative thinking as a transdiagnostic bridge between childhood trauma and depression: Integrating emotion regulation flexibility, stress sensitization, and neurocognitive mechanisms

**DOI:** 10.3389/fpsyt.2026.1941842

**Published:** 2026-10-19

**Authors:** Chen Yang

**Affiliations:** College of Marxism, Nanjing University of Science and Technology, Nanjing, China

**Keywords:** childhood trauma, cognitive vulnerability, depression, emotion regulation, repetitive negative thinking, stress sensitization, transdiagnostic mechanisms

## Abstract

Childhood trauma is a major risk factor for depression; however, not every affected individual develops clinically significant depressive symptoms. This underscores the importance of identifying transdiagnostic mechanisms that account for vulnerability and resilience. Repetitive negative thinking (RNT), which includes both rumination and worry, has recently been framed as a more general cognitive process that may help clarify how early adverse experiences contribute to the emergence and persistence of depression. This narrative review integrates current evidence regarding RNT as a possible transdiagnostic link connecting childhood trauma to depression, with particular focus on its interactions with emotion regulation flexibility, stress sensitization, and neurocognitive processes underlying self-referential cognition, cognitive control, and threat processing. Findings from developmental psychopathology, cognitive neuroscience, and affective science indicate that childhood trauma is often associated with persistent RNT, diminished regulatory flexibility, and increased sensitivity to stress—factors that are each independently related to depressive symptoms. Emerging evidence also suggests that these mechanisms are likely interrelated and may collectively increase susceptibility to depression across diagnostic categories. Nonetheless, substantial variability in trauma features, developmental timing, outcome measures, and study designs along with the dominance of cross-sectional research, limits the ability to draw definitive causal conclusions. Taken together, the existing literature indicates the value of a transdiagnostic perspective while highlighting important methodological and conceptual shortcomings. Future longitudinal and mechanistic studies are required to specify causal pathways and to test whether interventions aimed at RNT and related regulatory processes can meaningfully enhance the prevention and treatment of depression associated with trauma.

## 1 Introduction

Childhood trauma constitutes a major global public health issue, with significant and lasting effects on mental health throughout the lifespan. Adverse childhood experiences, including emotional, physical, and sexual abuse, as well as neglect, have repeatedly been linked to greater susceptibility to a broad spectrum of psychiatric disorders, especially depression (1, 2). In addition to its high prevalence, childhood trauma is marked by considerable developmental consequences, affecting stress reactivity, emotional processing, cognitive performance, and interpersonal adjustment. Epidemiological findings suggest that exposure to adversity in early life increases the likelihood of depressive symptoms and major depressive disorder; however, the connection between childhood trauma and subsequent depression is multifaceted and cannot be reduced to a straightforward causal chain (3). Notably, many people who experience childhood adversity do not develop depression, while others develop ongoing and markedly severe depressive symptoms, indicating that intermediate psychological and neurobiological processes likely influence individual vulnerability and resilience (4). Depression that develops after childhood trauma shows substantial heterogeneity in how symptoms present, how severe they are, how patients respond to treatment, and what underlying mechanisms are involved. People with similar trauma histories can nonetheless show strikingly different clinical courses, underscoring the need to move beyond focusing solely on trauma exposure and instead pinpoint the specific processes that mediate the shift from early adversity to depressive psychopathology (5). Existing frameworks have proposed multiple mechanisms, such as dysfunction of the hypothalamic–pituitary–adrenal (HPA) axis, inflammation, difficulties in emotion regulation, negative cognitive schemas, and changes in neural circuits that support threat processing and reward sensitivity (6). Even so, these mechanisms probably interact in dynamic ways rather than acting independently, and their relative impact is likely to differ depending on features of the trauma, developmental timing, genetic vulnerability, and the surrounding environmental context.

Conventional psychiatric classification systems have mainly treated depression as a distinct diagnostic category; however, growing evidence indicates that many psychological and biological mechanisms involved in depression overlap with those in other mental disorders (7). As a result, disorder-specific models may offer only a limited account of trauma-related depression when they emphasize symptom groupings rather than shared mechanisms. This shortcoming has helped drive the development of transdiagnostic frameworks, which seek to pinpoint common cognitive, emotional, and neurobiological processes that increase vulnerability across diagnostic boundaries (8). From this standpoint, processes such as maladaptive cognitive processing, reduced emotion regulation, and heightened stress sensitivity are understood as dimensional mechanisms that may support the onset and persistence of depressive symptoms in individuals with a wide range of clinical presentations. Among these transdiagnostic mechanisms, repetitive negative thinking (RNT) has attracted growing interest as a potentially key cognitive process through which early adversity may relate to depression. RNT denotes a style of persistent, repetitive, and hard-to-control negative thoughts that can encompass both depressive rumination and anxiety-related worry (9). While rumination has long been framed as a depression-specific cognitive vulnerability involving repeated attention to negative emotions and their consequences, more expansive accounts of RNT indicate that repetitive thinking may function as a common mechanism spanning several forms of psychopathology (10, 11). This distinction is important because childhood trauma may not only increase depressive rumination but may also contribute to a broader tendency toward perseverative cognitive processing characterized by persistent negative self-focused thought and difficulties disengaging from distressing cognitive content (12). Recent neuroimaging evidence provides further support for this broader conceptualization. In an fMRI study, worry and rumination elicited overlapping neural representations, supporting the view that these processes share a common neural basis within the broader construct of RNT (13). Recent theoretical and empirical developments further indicate that RNT may operate in conjunction with emotion regulation flexibility (ERF), stress sensitization, and neurocognitive processes to shape depressive vulnerability after childhood trauma. ERF, rather than regulation failure alone, may be a key determinant of adaptive functioning, because individuals need to choose and adjust strategies in response to contextual demands (14). Likewise, stress sensitization frameworks suggest that early adversity can modify how people respond to later stressors, which may heighten the risk of depressive episodes in subsequent life (15). Neurocognitive pathways that involve reduced cognitive control, altered self-referential processing, and attentional biases toward negative information may also help explain why maladaptive thought patterns persist (16, 17). Nonetheless, notwithstanding substantial advances, the current body of evidence remains heterogeneous, and the exact causal links among trauma exposure, RNT, regulatory processes, and depression still call for additional clarification through longitudinal and mechanistic studies.

Therefore, this narrative review is intended to offer a transdiagnostic synthesis of the current evidence on RNT as a potential bridging mechanism linking childhood trauma to depression. Specifically, we consider how RNT relates to ERF, stress sensitization, and neurocognitive dysfunction in ways that increase risk for depressive outcomes. By bringing together insights from developmental psychopathology, cognitive neuroscience, and affective science, this review aims to go beyond symptom-based accounts and to emphasize shared mechanisms that could guide future research and the design of more individualized prevention and intervention strategies.

## 2 Literature search strategy and methodology

This narrative review aimed to deliver a comprehensive and critical synthesis of the existing evidence regarding RNT as a transdiagnostic mechanism that links childhood trauma to depression. While this review was not carried out as a systematic review or meta-analysis, a structured literature search was conducted to improve transparency, reproducibility, and the overall scope of the evidence. Searches were performed in PubMed/MEDLINE, Scopus, Web of Science Core Collection, PsycINFO, and Google Scholar, and were supplemented by manual screening of the reference lists of relevant reviews and eligible articles to locate additional studies. The search encompassed publications from January 2000 through June 2026, with the final search completed in June 2026, highlighting the considerable growth of research during this period on transdiagnostic psychopathology, developmental trauma, and cognitive-affective mechanisms. Search strategies used controlled vocabulary, where available, together with free-text keywords connected by Boolean operators. Representative search terms comprised childhood trauma, early life adversity, adverse childhood experiences, depression, major depressive disorder, RNT, rumination, perseverative cognition, emotion regulation, ERF, stress sensitization, cognitive control, executive function, self-referential processing, transdiagnostic, and neurocognitive dysfunction. Keywords and search strings were repeatedly refined across databases to enhance both sensitivity and specificity.

Given the inherently multidisciplinary scope of the review, a narrative synthesis was selected rather than a systematic review or meta-analysis. The literature spans developmental psychopathology, cognitive and affective neuroscience, stress biology, emotion regulation, and psychiatry, with substantial heterogeneity in study designs, populations, trauma characteristics, RNT-related constructs, neurocognitive measures, and clinical outcomes. This cross-domain heterogeneity limits the suitability of quantitative pooling and makes an integrative narrative approach more appropriate for examining convergent mechanisms, discrepancies, and conceptual relationships across levels of analysis. The electronic searches of PubMed/MEDLINE, Scopus, Web of Science Core Collection, PsycINFO, and Google Scholar identified 1,352 records. After removal of 318 duplicate records, 1,034 records remained for title and abstract screening. Following title and abstract screening, 124 publications underwent full-text assessment. After full-text evaluation and backward citation tracking, 62 publications were retained as the evidence base for the narrative synthesis. These included primary studies, systematic reviews, meta-analyses, theoretical articles, and mechanistic studies relevant to the review objectives. Among the retained literature, 19 primary studies directly informed the core neurocognitive and intervention-focused evidence synthesis and are summarized in the main evidence tables.

Eligible publications included peer-reviewed original studies, longitudinal cohort studies, randomized clinical trials, systematic reviews, meta-analyses, and highly influential theoretical articles that investigated associations among childhood trauma, RNT or related cognitive processes, emotion regulation, stress-related mechanisms, neurocognitive functioning, and depressive outcomes. Relevant preclinical studies were also considered when they offered mechanistic insights with potential translational relevance. Conference abstracts, editorials, commentaries, dissertations, unpublished reports, and studies that lacked sufficient methodological detail or relevance to the review objectives were excluded. Only articles published in English were included. Titles and abstracts identified via the electronic search were first screened for relevance, and studies that appeared potentially eligible were then assessed through full-text review. Further publications were located by conducting backward citation searches of key reviews and seminal articles. Priority was given to recent high-quality evidence, especially longitudinal investigations, systematic reviews, meta-analyses, and mechanistic studies, although landmark publications were also included when they made substantial contributions to the field’s conceptual development. The findings were synthesized narratively, with particular emphasis on integrating evidence across developmental psychopathology, affective science, cognitive neuroscience, and psychiatry to identify convergent mechanisms, account for discrepancies across studies, clarify likely sources of heterogeneity, and develop an integrated transdiagnostic framework connecting childhood trauma with depression through RNT, ERF, stress sensitization, and neurocognitive dysfunction. Aligned with the aims of a narrative review, no formal risk-of-bias assessment or quantitative meta-analysis was conducted.

## 3 Childhood trauma as a developmental risk factor for depression

Childhood trauma is increasingly recognized as a developmental vulnerability factor that affects emotional, cognitive, and biological adaptation, rather than functioning as a direct, deterministic cause of depression (18). While epidemiological studies consistently show a link between early adversity and later depressive outcomes, substantial variability is observed across trauma-exposed individuals in symptom presentation, clinical course, and biological responses. This heterogeneity indicates that childhood trauma alters depression risk through interacting pathways that involve stress regulation, emotional processing, cognitive appraisal, and environmental adaptation (**Table 1****).** Consequently, viewing childhood trauma as a developmental risk factor entails accounting for trauma features, the timing of development, and individual differences in vulnerability and resilience (1, 22, 24, 25).

**Table 1:** Developmental, psychological, and neurobiological characteristics of childhood trauma associated with increased vulnerability to depression.

Trauma subtype/adversity examined	Main psychological consequences	Neurobiological alterations	Association with depression vulnerability	Study
Emotional abuse, physical abuse, sexual abuse, and household dysfunction (cumulative adverse childhood experiences)	Increased psychosocial vulnerability, maladaptive coping patterns, emotional distress, and elevated psychiatric burden	Suggested cumulative effects of early adversity on stress-related biological systems	Greater exposure to multiple ACE categories was associated with a graded increase in depression risk, suicidal behavior, and other adverse health outcomes	(1)
Childhood sexual and physical abuse	Increased stress sensitivity and vulnerability to mood and anxiety symptoms	Persistent alterations in hypothalamic–pituitary–adrenal (HPA) axis activity and autonomic stress responses; enhanced CRF-related stress reactivity	Childhood abuse was associated with increased stress responsivity, particularly among individuals with major depression, suggesting a possible pathway linking early adversity to mood disorders	(19)
Child maltreatment, including abuse and neglect	Increased internalizing symptoms, emotional dysregulation, and behavioral difficulties	Altered cortisol regulation, including elevated cortisol levels and disrupted diurnal cortisol patterns in maltreated children with internalizing symptoms	Maltreatment-related neuroendocrine alterations were associated with increased vulnerability to psychopathological outcomes, including depressive symptoms	(20)
Moderate-to-severe childhood maltreatment, including emotional neglect and sexual abuse	Increased vulnerability to stress-related psychopathology despite absence of current psychiatric illness	Altered HPA-axis responsivity characterized by reduced ACTH and cortisol responses following psychosocial stress	Childhood maltreatment was associated with persistent stress-system alterations that may contribute to later vulnerability to depression and anxiety	(21)
Documented childhood physical abuse, sexual abuse, and neglect	Increased psychiatric vulnerability, earlier onset of psychopathology, and greater psychiatric comorbidity	Not directly examined; longitudinal findings indicate developmental consequences of maltreatment exposure	Childhood abuse and neglect were associated with increased risk of major depressive disorder in young adulthood, particularly physical abuse and neglect	(22)
Parental maltreatment, including abuse and neglect	Increased vulnerability to chronic emotional difficulties and maladaptive developmental trajectories	Not primarily assessed; focused on measurement of childhood maltreatment and its relationship with depression	Retrospectively assessed parental maltreatment was associated with increased risk of chronic depressive episodes, supporting long-term links between early adversity and depression	(23)
Multiple childhood adversities, including physical abuse, sexual abuse, neglect, family violence, parental psychopathology, and dysfunction	Increased emotional vulnerability and broader psychiatric susceptibility	Not directly assessed; emphasized cumulative and interactive effects of adversity	Childhood adversities were strongly associated with first onset of mood and other psychiatric disorders, although associations were not fully specific to depression	(24)
Childhood maltreatment (physical, sexual, and emotional abuse, neglect), parental dysfunction, loss, and socioeconomic adversity	Impaired emotional adaptation, increased psychological vulnerability, and developmental risk	Not directly assessed; highlighted the importance of developmental timing and cumulative adversity	Childhood adversities were associated with increased risk of psychiatric disorder onset during adolescence, including mood disorders	(25)
Childhood maltreatment assessed using the Childhood Trauma Questionnaire	Increased threat sensitivity, negative affective processing, and emotional vulnerability	Increased amygdala responsiveness to threat-related stimuli; reduced gray matter volume in hippocampus, insula, orbitofrontal cortex, anterior cingulate cortex, and caudate	Trauma-related neural alterations resembled abnormalities observed in depression and PTSD, suggesting potential mechanisms linking maltreatment to emotional disorders	(26)
Childhood trauma, particularly physical abuse, among individuals with major depression	Altered processing of negative emotional information and trauma-related depressive phenotypes	Increased amygdala responsiveness to sad facial expressions, particularly in individuals with physical abuse histories	Findings suggested that childhood trauma may influence neural mechanisms underlying depression and contribute to biologically distinct depression subtypes	(27)
Adverse childhood experiences, including family instability, poverty, parental separation, and cumulative ACE exposure	Increased cumulative psychosocial vulnerability and impaired long-term adaptation	Not directly assessed; emphasized clustering and cumulative effects of adversity	Higher ACE burden and specific adversity patterns were associated with increased premature mortality, supporting long-term consequences of childhood adversity	(28)
Childhood poverty and cumulative socioeconomic adversity	Increased psychological stress, reduced adaptive capacity, and vulnerability to emotional dysregulation	Elevated overnight cortisol levels, altered cardiovascular stress reactivity, and impaired stress regulation	Chronic socioeconomic adversity during childhood may increase depression vulnerability through persistent dysregulation of stress-response systems	(29)

### 3.1 Types of childhood trauma

Childhood trauma comprises several types of adverse experiences, including emotional abuse, physical abuse, sexual abuse, emotional neglect, physical neglect, and broader adverse childhood experiences involving family dysfunction. Epidemiological research has consistently suggested that trauma exposure seldom occurs in isolation. Rather, different forms of adversity often co-occur within the same developmental context. As a result, cumulative exposure and the overall level of adversity may serve as more powerful predictors of later psychiatric vulnerability than any single trauma category alone (1, 24, 25). Distinct categories of trauma can shape depression vulnerability through partly overlapping yet distinguishable mechanisms. Experiences involving threat, including physical and sexual abuse, may increase threat sensitivity, heighten emotional reactivity, and sustain activation of stress-related systems. In contrast, deprivation-related experiences, particularly emotional neglect, may disrupt emotional learning, interfere with interpersonal development, and hinder the development of adaptive emotion regulation strategies. Nevertheless, these differences should be interpreted with caution because abuse and neglect often occur together. It therefore remains difficult to disentangle their separate effects within clinical samples (22, 24). Prospective findings show that childhood maltreatment is associated with a higher likelihood of depressive disorders in adulthood. However, the strength of these associations differs according to trauma type and how outcomes are defined. For instance, longitudinal results indicate that physical abuse and neglect are particularly important predictors of later major depressive disorder (22). However, the association between specific trauma categories and depression may also be shaped by concurrently occurring adversities and methodological variations in assessment. Likewise, retrospective evaluations of parental maltreatment have revealed associations with prolonged depressive episodes. This finding is consistent with the idea that early relational adversity may contribute to enduring vulnerability to depression (23). Neurobiological research further indicates that distinct forms of childhood trauma may affect emotional processing systems implicated in depression. Individuals with higher levels of childhood maltreatment have shown altered amygdala reactivity to emotional stimuli and structural differences in brain regions involved in stress regulation, memory, and cognitive control, including the hippocampus and prefrontal-related areas (26). However, these changes should not be interpreted as depression-specific neural signatures. Comparable patterns have been documented across several stress-related psychiatric disorders.

### 3.2 Developmental timing

The timing of childhood trauma during development is an important determinant of later psychological outcomes. Early developmental stages feature rapid maturation of neural circuits, stress-response systems, and emotion-regulation abilities; thus, adversity that occurs during these periods may disrupt typical developmental trajectories. Still, the current body of evidence does not support a straightforward model in which trauma exposure within a particular age window invariably leads to depression. Rather, developmental timing shapes individual pathways through its interactions with trauma severity, duration, cumulative exposure, and subsequent environmental experiences (24, 25). Adversity in early childhood can influence attachment processes, emotional learning, and the emergence of regulatory capacities, whereas adversity during adolescence may interact with ongoing neurodevelopmental changes and escalating social demands. Large epidemiological studies suggest that experiences of adversity during childhood are linked to a higher likelihood of the onset of psychiatric disorders across developmental stages; however, these links depend on the type, number, and context of adverse experiences, rather than on the age at which exposure occurs alone (24, 25). Importantly, developmental timing should be evaluated alongside chronicity. Repeated exposure to adverse environments may yield effects that differ from those of singular traumatic events, since ongoing stress can continuously tax developing regulatory systems. Nevertheless, variability in outcomes shows that exposure length by itself cannot fully account for why some individuals develop depression while others maintain adaptive functioning.

### 3.3 Stress sensitization hypothesis

The stress sensitization hypothesis offers a crucial framework for understanding how childhood trauma may heighten the risk of depression. According to this model, early adversity can recalibrate stress-response systems, thereby increasing sensitivity to subsequent stressors and lowering the threshold for depressive episodes. Experimental research indicates that childhood maltreatment is linked to enduring changes in HPA axis regulation and autonomic stress responses, though the direction and size of these effects differ across populations (19–21). The stress sensitization framework also extends beyond peripheral neuroendocrine and autonomic systems to stress-sensitive brain circuits. In particular, the hippocampus may undergo persistent structural and functional adaptations following early adversity, potentially altering its response to subsequent stressors. Reduced hippocampal volume has been proposed as one neurobiological mechanism contributing to stress sensitization to depression following childhood trauma, suggesting that trauma-related hippocampal alterations may increase vulnerability to later stress exposure (30). Early findings suggested that people with histories of childhood abuse may show heightened endocrine and autonomic reactions to stress, especially those who currently report depressive symptoms, consistent with sustained sensitization of stress-related systems (19). In contrast, other studies have reported diminished adrenocorticotropic hormone and cortisol responses in adults with childhood maltreatment histories, even when no present psychopathology is detected, implying that chronic adversity may also produce adaptive or compensatory modifications in stress regulation (21). Findings from maltreated children further substantiate the existence of altered cortisol regulation, especially among those with internalizing symptoms (20). At first glance, these seemingly inconsistent results point to a key source of heterogeneity: childhood trauma does not appear to elicit a single, uniform biological response pattern. Rather, changes in the stress-response system may vary according to developmental stage, features of the trauma, current mental health status, and individual adaptation processes. Accordingly, childhood adversity may affect the flexibility and regulation of stress-response systems, rather than simply leading to persistent hyperactivation or suppression. The stress sensitization framework also offers a conceptual route that links childhood trauma to transdiagnostic cognitive mechanisms. Dysregulated stress responses may heighten negative emotional processing, strengthen attentional biases toward threat, and impair the ability to disengage from distressing thoughts. These mechanisms may, in turn, promote RNT, which may serve as a cognitive link between trauma exposure and depressive symptoms.

### 3.4 Why only some individuals develop depression

Although there is strong evidence that childhood trauma is associated with a higher risk of depression, many people who experience childhood adversity do not go on to develop depressive disorders. This finding suggests that exposure to trauma functions as a vulnerability factor rather than a sufficient cause of depression. Variation in genetic susceptibility, the availability of social support, coping strategies, emotion regulation ability, and later life experiences could help explain these different developmental trajectories. One account of this heterogeneity is that childhood trauma may affect depression indirectly through intermediary psychological processes. Instead of directly generating depressive symptoms, adversity may heighten the probability of maladaptive cognitive and emotional patterns, such as deficient emotion regulation, negative self-referential processing, and RNT. These processes may influence whether trauma-related emotional reactions endure and thereby contribute to the emergence of depressive symptoms. Neurocognitive results indicate that trauma exposure may produce a state characterized by heightened emotional vulnerability, rather than representing a fixed pathological condition. Altered amygdala responses and trauma-related neural differences found in people with childhood adversity point to changes in emotional information processing; however, these patterns differ depending on trauma characteristics and clinical presentation (26, 27). The observation that amygdala responses vary by trauma subtype implies that childhood trauma could foster specific routes toward depression, rather than reflecting a single biological phenotype (27). From a translational standpoint, these findings highlight the need to identify modifiable mechanisms that clarify why some individuals exposed to trauma go on to develop depression. Clinically, concentrating solely on trauma exposure may have limited value, because adversity alone does not determine psychiatric outcomes. Instead, addressing intermediate processes such as RNT, ERF, and stress adaptation may offer a more precise framework for understanding and potentially lessening, depression vulnerability following childhood trauma.

## 4 RNT: beyond traditional rumination

RNT has emerged as an important construct in present-day psychopathology research, in part because it reflects a typical cognitive process that underlies ongoing emotional distress across multiple psychiatric disorders. Historically, depression-focused work has mostly emphasized rumination as a defining characteristic of depression; however, a growing body of evidence indicates that repetitive negative thought goes beyond depressive rumination and instead reflects a wider cognitive style characterized by repetitive, hard-to-control, and negatively valenced thinking. By adopting this broader framing, researchers can investigate shared cognitive mechanisms across diagnostic groups rather than concentrating only on disorder-specific symptoms (9, 31). Recent neuroimaging research has also provided empirical support for the neural correlates of RNT. In individuals with major depressive disorder, trait RNT has been associated with functional connectivity patterns that become particularly evident during negative-thinking states (32). RNT is distinguished not primarily by the specific content of thoughts, but by the process by which thoughts are produced and sustained. People who engage in RNT repeatedly scrutinize negative experiences, potential causes, consequences, or implications without necessarily arriving at effective resolution. This recurring cognitive activity is distinct from adaptive reflection or problem solving because it is more likely to sustain negative affect, diminish cognitive flexibility, and disrupt coping responses that are effective. Critically, RNT does not suggest that negative thoughts are inaccurate; instead, the maladaptive aspect lies in the repetitive, persistent way in which these thoughts are processed (10).

### 4.1 Definition of RNT

RNT denotes a pattern of repetitive, protracted, and hard-to-disengage negative thinking that is linked to ongoing emotional suffering. The construct was proposed to unify cognitive processes that had previously been examined separately, especially depressive rumination and worry related to anxiety. Thus, rumination and worry can be conceptualized as phenomenologically distinct manifestations of the broader RNT construct rather than as separate transdiagnostic processes. A defining characteristic of RNT is that the same cognitive process can manifest across different emotional disorders, notwithstanding differences in the particular themes or concerns under consideration (9). A key aspect of conceptualizing RNT is distinguishing between thought content and thought process. Conventional diagnostic frameworks typically prioritize the topics of cognition—for example, sadness-related thoughts in depression or threat-related thoughts in anxiety. By contrast, RNT emphasizes the repetitive manner of cognitive processing itself. From this process-focused viewpoint, persistent negative thinking may reflect a common vulnerability mechanism that helps explain emotional dysregulation across disorders (31). The Perseverative Thinking Questionnaire was created to measure RNT as a content-independent construct. This method examines fundamental features of repetitive thinking, such as repetitiveness, intrusiveness, difficulty disengaging, and perceived uncontrollability. Validation studies have shown that RNT can be assessed independently of particular psychiatric diagnoses, underscoring its value as a transdiagnostic cognitive construct (31). Recent longitudinal neuroimaging evidence has further shown that RNT-related neural network dynamics can change following psychological and pharmacological treatment, highlighting the potential malleability of neural processes underlying RNT (33).

### 4.2 Rumination vs worry vs perseverative cognition

Rumination and worry represent two well-studied manifestations of RNT. Although they are often linked to different disorders, both share key features, including recurrent cognitive engagement, a negative emotional tone, and difficulty disengaging cognitively. Rumination is usually understood as a process characterized by repeated attention to negative emotions and to their causes, meanings, and consequences, whereas worry typically involves repetitive thoughts about potential future threats or negative outcomes (34, 35). Despite differences in temporal focus, rumination and worry overlap conceptually to a significant degree. Rumination is frequently directed toward past or present distress, while worry is largely oriented to the future; nonetheless, both can operate as maladaptive cognitive responses that sustain emotional arousal rather than promoting effective resolution. Meta-analytic results show strong connections between rumination and worry, indicating that these constructs may reflect partially overlapping expressions of a broader repetitive negative cognitive process (36). Perseverative cognition provides a related theoretical framework that emphasizes the sustained activation of cognitive representations tied to stress, threat, or negative experiences. The notion of perseverative cognition further broadens this framework by highlighting the sustained activation of cognitive representations tied to stress, threat, or negative experiences. Under the perseverative cognition hypothesis, ongoing cognitive engagement with negative information can preserve emotional and physiological responses even when there is no immediate external challenge. This model offers a theoretical foundation for explaining how RNT and related repetitive cognitive processes may support continued distress over time (37). Consequently, instead of viewing rumination and worry as separate symptoms that belong exclusively to depression and anxiety, respectively, RNT offers a single framework for investigating shared cognitive processes that drive emotional difficulties across different conditions.

### 4.3 Why RNT is considered transdiagnostic

The transdiagnostic importance of RNT is supported by evidence indicating that it occurs across diverse forms of psychopathology, including depressive disorders, anxiety disorders, post-traumatic stress disorder, eating disorders, and other emotional disorders. Rather than serving as a symptom specific to a single disorder, RNT appears to operate as a shared cognitive process linked to the persistence of symptoms, their severity, and diminished recovery (9, 38). In depression, ruminative forms of RNT have repeatedly been associated with extended negative mood states, compromised problem-solving ability, lower motivation, and a greater likelihood of symptom persistence. Experimental findings also indicate that ruminative RNT can amplify negative affect and constrain adaptive cognitive responses, thereby supporting the interpretation that RNT contributes to the maintenance of depressive symptoms rather than merely representing an emotional outcome of depression (39). Likewise, worry, as a future-oriented form of RNT, has been recognized as a key factor that sustains anxiety disorders. While worry may initially seem to reflect efforts to anticipate and handle possible difficulties, ongoing worry can prove harmful by keeping attention locked on perceived threats and by impairing emotional processing. The existence of comparable repetitive cognitive patterns in both depression and anxiety further indicates the usefulness of investigating RNT as a shared mechanism, rather than a phenomenon limited to particular diagnoses (35, 36). Accordingly, from a transdiagnostic standpoint, RNT may function as an important cognitive process through which negative emotional states are extended and begin to reinforce themselves. This framing is consistent with current dimensional models of psychopathology, which highlight mechanisms that operate across conventional diagnostic boundaries.

### 4.4 Cognitive mechanisms maintaining RNT

Multiple cognitive mechanisms are involved in the onset and continued presence of RNT. A key mechanism is compromised attentional disengagement from negative information. People with higher levels of RNT may find it hard to shift attention away from emotionally significant thoughts or stimuli, which can lead to sustained processing of negative material. The impaired disengagement account suggests that ongoing negative thinking is maintained, at least in part, by lower cognitive flexibility and difficulty redirecting attention to other information (40). Another important mechanism concerns the degree of abstraction at which negative experiences are processed. Watkins argued that maladaptive repetitive thinking is frequently characterized by abstract, evaluative processing, where individuals attend to overarching meanings, consequences, and self-assessments rather than to specific contextual details. This abstract mode of processing may undermine effective problem solving and heighten emotional distress by preserving broadly construed negative interpretations (10). Metacognitive beliefs also make an important contribution to sustaining RNT. People may come to hold convictions that repetitive thinking is helpful, required, or protective—for example, the belief that ongoing analysis will produce better understanding or help avert future problems. While such beliefs can promote continued engagement in repetitive thinking, they can paradoxically strengthen uncontrollable cognitive loops (41). Recent ecological momentary assessment research has shown that RNT can be reliably captured in daily life and that higher levels of daily-life RNT predict subsequent psychopathological symptoms, including depression and anxiety, beyond questionnaire-based measures (42). Moreover, impairments in cognitive flexibility and in adaptive emotion regulation may help explain why RNT persists. Struggles with switching viewpoints, producing alternative interpretations, and disengaging from negative thoughts may enable repetitive cognitive patterns to maintain themselves. Importantly, these mechanisms reflect broad cognitive processes rather than disorder-specific abnormalities, which supports viewing RNT as a transdiagnostic pathway implicated in emotional dysregulation. Overall, RNT offers a wider theoretical framework than conventional rumination models by highlighting a common cognitive process marked by repetitive, rigid, and negatively oriented thought. Gaining insight into these cognitive mechanisms is crucial for assessing how RNT works alongside other vulnerability factors in the emergence and persistence of depression and related emotional disorders.

## 5 ERF: a missing link between trauma and depression

### 5.1 Emotion dysregulation versus ERF

Childhood trauma may affect the risk of depression not only by heightening emotional reactivity, but also by changing the flexibility with which emotional responses are managed across varying contexts (14, 43). Repeated exposure to unpredictable, threatening, or uncontrollable environments may limit the development of regulatory responses that are sensitive to context, thereby making it harder to choose, shift, or disengage from regulatory strategies as emotional demands change (14, 43). This distinction is especially relevant to RNT, since persistent RNT may, in part, reflect difficulties in redirecting attention from negative internal states or in adjusting regulatory responses when RNT stops being effective (44). Accordingly, ERF offers a conceptual link among early adversity, emotion regulation, and RNT: reduced flexibility in regulatory processes may extend negative affect and increase reliance on RNT, which in turn can contribute to vulnerability to depression (43, 44).

Emotion dysregulation has long been conceptualized as diminished ability to modulate emotional reactions, which often leads to emotional instability, sustained negative affect, and maladaptive coping. Although this deficit-focused viewpoint has considerably improved understanding of depression, it offers limited explanation for why people with similar emotional difficulties often show substantially different adaptive outcomes. As a result, more recent theoretical accounts have redirected attention from emotional deficits to ERF, defined as the capacity to adjust regulatory responses in line with shifting situational requirements rather than depending on fixed regulatory routines (14). In contrast to traditional models that categorize emotion regulation strategies as either adaptive or maladaptive, ERF argues that no single strategy is universally effective. For instance, cognitive reappraisal may promote adaptation in situations that can be controlled, while acceptance or short-term suppression may be better suited when immediate emotional demands cannot be changed. Therefore, the effectiveness of a regulatory response depends mainly on how well it fits the context, rather than on the strategy itself (43). This process-focused viewpoint has redirected research away from merely identifying preferred strategies toward clarifying how individuals appraise environmental signals, choose regulatory options, and revise them when circumstances shift. Notably, emotion dysregulation and ERF should not be treated as opposing constructs. Individuals may have access to multiple regulation strategies while still showing restricted flexibility in selecting them or switching among them. In contrast, effective emotional functioning involves ongoing monitoring of contextual feedback, judging whether regulatory efforts have been successful, and promptly modifying responses that prove ineffective. By highlighting adaptive variability rather than reliance on a rigid strategy, ERF offers a wider conceptual basis for understanding emotional adaptation and provides a valuable starting point for investigating cognitive vulnerability mechanisms that contribute to depression (45).

### 5.2 Flexible strategy selection

ERF goes beyond simply having multiple regulatory strategies; it also highlights the capacity to choose the most suitable strategy for a particular context. Contemporary models propose that effective emotional adaptation relies on keeping a varied regulatory repertoire and using different strategies based on situational controllability, emotional intensity, interpersonal requirements, and personal objectives, rather than consistently depending on a single preferred response (46). As a result, regulatory success is increasingly understood as a dynamic process of decision-making instead of the routine application of strategies that are typically regarded as adaptive. Experimental studies have shown that individuals can adjust their regulatory preferences as contextual demands shift, though the extent of this ability differs markedly between people (47). For instance, cognitive reappraisal can be beneficial when stressful situations are controllable because it supports cognitive restructuring, whereas attentional distraction may yield stronger short-term benefits during highly intense emotional states that temporarily surpass cognitive resources. Similarly, acceptance can support adjustment when stressors cannot be easily changed. Taken together, these findings suggest that psychological functioning is determined less by how often specific strategies are used than by how precisely regulatory responses are matched to situational features. Notably, selecting strategies flexibly depends on ongoing monitoring of regulatory effectiveness. If a strategy chosen at first does not lessen emotional distress or disrupts current goals, adaptive regulation entails stepping away from that strategy and adopting an alternative approach (48). This continuous appraisal differentiates regulatory flexibility from mere strategy availability and underscores the role of cognitive adaptability in preserving emotional equilibrium. This viewpoint offers a process-oriented framework for explaining emotional functioning and provides an important conceptual basis for the later sections that consider how regulatory flexibility may interact with RNT in depression.

### 5.3 Context sensitivity

Sensitivity to context is regarded as a central element of ERF, since successful regulation relies on correctly determining the requirements of a particular situation before choosing a suitable course of action. Rather than presuming that diminishing negative emotion is invariably advantageous, current models argue that effective, adaptive regulation depends on judging whether the emotional response should be sustained, weakened, or even strengthened in order to align with present objectives (49). This view undermines older ways of categorizing regulatory strategies as either uniformly adaptive or uniformly maladaptive and instead highlights how situational features interact with regulatory behavior. Context-sensitive regulation entails combining several types of information, including the intensity of the emotion, the degree to which the stressor is controllable, the social and interpersonal repercussions, and the cognitive resources that are available. For instance, continuing to express emotion can support social communication in affirming settings, while brief emotional suppression may be beneficial when circumstances call for swift decision-making or conflict management. Consequently, the same regulatory approach can lead to either beneficial or unfavorable outcomes depending on the circumstances in which it is implemented (50). This dynamic viewpoint underscores the value of flexible evaluation rather than strict commitment to favored coping styles. Recent conceptual frameworks also indicate that sensitivity to context is an ongoing process, not a one-time choice. People continually assess feedback from their surroundings and adjust their regulatory efforts when conditions shift or when initial strategies fail to work. This kind of iterative recalibration facilitates emotional adaptation and lowers the risk of sustained emotional activation (51). Thus, context sensitivity is a crucial mechanism through which ERF fosters psychological adjustment and offers an essential conceptual foundation for explaining why regulatory processes may vary greatly among individuals even when they face comparable emotional challenges.

### 5.4 Failure of emotional recovery

A crucial, yet frequently underappreciated, component of ERF is the ability to rebound effectively following emotional activation. Even when individuals show broadly similar initial emotional responses to stressful events, they can differ markedly in how quickly and how completely emotional, cognitive, and physiological reactions return to baseline. Accordingly, contemporary models separate emotional reactivity from emotional recovery, suggesting that sustained psychological distress may depend more on extended recovery—rather than on elevated initial reactivity alone—than previously thought (52). Emotional recovery is increasingly understood as an active regulatory undertaking that involves disengaging from affectively salient experiences, replenishing cognitive resources, and refocusing on current goals. Delayed recovery may stem from the continued use of attentional resources for emotionally relevant information, diminished cognitive flexibility, or ongoing engagement in maladaptive appraisal processes; each of these factors can disrupt adaptive functioning (53). Accordingly, effective regulation should not be judged solely by immediate emotional reactions, but also by the ability to stop those reactions once environmental demands have eased. From the standpoint of flexibility, successful recovery relies on continuously tracking emotional feedback and adjusting regulatory actions when current strategies no longer meet their intended goals. People who stay confined to a single regulatory mode may show extended emotional activation even after contextual demands change. By contrast, flexible reconfiguration promotes the return of emotional balance and bolsters resilience after stressful experiences (54). This contrast positions emotional recovery as a dynamic element of adaptive regulation and establishes an important conceptual bridge between regulatory flexibility and ongoing affective symptoms, thereby preparing the ground for investigating its relationship with RNT.

### 5.5 Interaction with RNT

ERF may serve as an important moderating mechanism that influences whether RNT remains a brief cognitive activity or instead evolves into a stable maladaptive pattern. Although RNT involves repetitive negative cognitions that are difficult to disengage from, its psychological effects may depend in part on a person’s capacity to flexibly manage the emotional states tied to these thoughts. People with greater regulatory flexibility may be better positioned to redirect attention, adjust interpretations, and choose alternative responses when repetitive negative thinking proves unproductive (55). Recent neuroimaging evidence further indicates that the adaptiveness of ERF is associated with depression and involves distinct prefrontal and temporoparietal neural mechanisms, highlighting the potential role of flexible regulation in depressive vulnerability (56). By contrast, lower flexibility may sustain RNT by restricting the ability to disengage from ineffective cognitive strategies. When individuals continue to use the same regulatory response despite limited effectiveness, negative thoughts may become progressively more self-perpetuating and continue to sustain emotional distress. This process aligns with wider models indicating that psychopathology is not necessarily driven by the mere presence of negative thoughts or emotions themselves, but instead by inflexible responses to internal experiences (57). Notably, the association between ERF and RNT ought to be viewed as dynamic rather than strictly unidirectional. RNT can hinder flexible regulation by constricting attentional focus, lowering sensitivity to contextual information, and increasing dependence on habitual cognitive responses. In contrast, limited ERF may diminish the ability to break RNT cycles and facilitate recovery from negative emotional states. Although empirical studies that directly test the interplay between ERF and RNT are still relatively scarce, combining these constructs may yield a more comprehensive framework for explaining individual differences in susceptibility to depression and emotional disorders (44).

## 6 Neurocognitive mechanisms underlying trauma-related RNT

Childhood trauma may heighten susceptibility to RNT via coordinated changes across multiple neurocognitive systems, rather than through a single pathological pathway. Emerging findings indicate that early adversity can shape how individuals detect emotionally salient information, manage attention, retrieve autobiographical memories, and process self-referential information. Such shifts may produce a cognitive context in which negative experiences receive excessive focus, remain inadequately placed within context, and are hard to disengage from. Notably, childhood trauma should not be treated as a direct cause of RNT or depression; instead, trauma-related neurocognitive alterations may function as vulnerability factors, with their influence contingent on developmental timing, trauma characteristics, biological susceptibility, and later environmental experiences. A key mechanistic framework centers on a mismatch between bottom-up emotional salience mechanisms and top-down regulatory networks. Childhood adversity has been linked to heightened responsiveness to threat-related cues and to altered limbic activity, alongside changes to prefrontal systems that support cognitive control and flexible regulation. This kind of imbalance could raise the probability that negative internal and external signals trigger extended RNT. Nevertheless, the findings are inconsistent, and not everyone who experiences trauma goes on to develop persistent RNT, indicating that these neurocognitive adaptations may represent both vulnerability and context-dependent patterns of response (**Table 2**).

**Table 2:** Neurocognitive mechanisms linking childhood trauma to repetitive negative thinking vulnerability.

Neurocognitive mechanism	Key findings	Potential relevance to RNT	Study
Threat-related attentional bias	Physically abused children showed delayed disengagement from angry facial expressions and enhanced attention to threat cues.	Persistent attention toward negative information may facilitate the maintenance of repetitive negative thoughts.	(58)
Altered threat attention processing	Maltreated children exhibited altered attention bias toward threatening stimuli, associated with abuse severity.	Increased sensitivity to threat may promote sustained negative cognitive focus.	(59)
Impaired cognitive control	Early-life stress was associated with poorer cognitive switching and altered activation in prefrontal and cingulate regions.	Reduced cognitive control may limit the ability to disengage from repetitive negative thoughts.	(60)
Executive dysfunction	Familial trauma exposure was associated with poorer working memory, inhibition, and interference control.	Executive difficulties may impair interruption of maladaptive cognitive cycles.	(61)
Overgeneral autobiographical memory	Maltreated children showed more overgeneral memories and greater negative self-representations.	Reduced memory specificity may contribute to persistent negative self-related thinking.	(62)
Limbic and structural brain alterations	Childhood maltreatment was associated with increased amygdala responsiveness and reduced gray matter in limbic-related regions.	Enhanced emotional salience may increase triggers for repetitive negative thinking.	(26)
Trauma-related amygdala reactivity	Childhood trauma was associated with increased amygdala responses to negative emotional stimuli in depressed individuals.	Heightened emotional responses may reinforce negative appraisal processes.	(27)
Reduced medial prefrontal cortex structure	Childhood emotional maltreatment was associated with reduced medial prefrontal cortex volume.	Altered prefrontal systems may compromise regulation of self-focused negative thoughts.	(63)
Altered self-referential network organization	Maltreatment was associated with altered network organization involving the cingulate cortex, precuneus, and insula.	Changes in self-processing networks may increase vulnerability to persistent self-focused cognition.	(64)
Enhanced neural threat reactivity	Children exposed to family violence demonstrated increased amygdala and anterior insula responses to angry faces.	Heightened threat appraisal may amplify negative internal experiences.	(65)
Hippocampal memory alterations	Childhood sexual abuse-related PTSD was associated with reduced hippocampal volume and impaired hippocampal activation.	Impaired contextual memory processing may contribute to persistence of trauma-related thoughts.	(66)

RNT, repetitive negative thinking; fMRI, functional magnetic resonance imaging; MRI, magnetic resonance imaging; mPFC, medial prefrontal cortex; ACC, anterior cingulate cortex.

### 6.1 Altered threat processing and attentional allocation after childhood trauma

One of the most consistent neurocognitive outcomes following childhood trauma is altered processing of threatening or emotionally negative information. Early adverse experiences may tune attentional systems toward heightened surveillance for potential danger. However, the direction of this bias can vary according to trauma severity, developmental stage, and psychopathological status. Among physically abused children, trauma exposure was associated with delayed disengagement from angry facial expressions, suggesting difficulty shifting attention away from threat-related cues (58). Likewise, maltreated children showed altered attentional responses to threatening stimuli, with associations between abuse severity and threat-related attentional processing (59).

Collectively, these findings suggest that trauma-related alterations in attention may continuously generate negative cognitive material, thereby contributing to RNT. When negative information is prioritized over neutral or positive alternatives, individuals may become more likely to repeatedly scrutinize perceived threats, interpersonal conflicts, or past adverse experiences. However, alterations in attention should not necessarily be considered uniformly maladaptive. In certain contexts, increased threat detection may represent an adaptive adjustment to unpredictable environments. Whether adaptive vigilance develops into maladaptive RNT may depend on whether these attentional patterns persist after the initial threat has disappeared.

### 6.2 Impaired cognitive control and reduced mental flexibility

Achieving effective regulation of repetitive thoughts depends on cognitive control mechanisms that enable people to redirect attention, suppress unhelpful cognitive material, and revise interpretations as circumstances change. Childhood trauma may undermine these regulatory abilities by disrupting prefrontal and cingulate systems that support cognitive flexibility and inhibitory control. Adolescents who experience early-life stress have demonstrated deficits in task-switching performance along with altered activation in brain regions involved in conflict monitoring and response control, including the anterior cingulate cortex and the inferior prefrontal cortex (60). Similarly, children who have experienced trauma show weaker executive functioning, including impairments in working memory, inhibition, and interference control, even when anxiety symptoms and socioeconomic factors are taken into account (61). These results suggest a plausible pathway by which RNT may become persistent: individuals may form negative interpretations but have a diminished ability to shift away from them or substitute them with alternative viewpoints. Still, executive dysfunction should not be viewed as a universal outcome of trauma. Differences in developmental resources, the presence of supportive relationships, and individual resilience factors may determine whether trauma exposure leads to persistent cognitive inflexibility.

### 6.3 Memory biases and maladaptive autobiographical processing

Memory processes constitute an additional route by which childhood trauma may contribute to RNT. Persistent negative thinking commonly involves repeated retrieval and elaboration of negative personal experiences; thus, changes in autobiographical memory may underlie the maintenance of these processes. Compared with nonmaltreated peers, maltreated children have shown reduced specificity in autobiographical memory and greater negative self-representations (62). These overgeneral memory patterns may promote wide-ranging negative judgments about the self, relationships, and future outcomes. Neurobiological findings further indicate that severe early adversity may alter hippocampal structure and function, which could affect the processing of contextual memory. Women who had childhood sexual abuse–related posttraumatic stress disorder exhibited a smaller hippocampal volume and reduced hippocampal activation during memory tasks (26). While these results mainly reflect trauma-related disorders rather than trauma exposure alone, they point to a potential pathway by which emotional memories may fail to be adequately integrated into the correct temporal and contextual frameworks. Accordingly, trauma-related memory changes may contribute to RNT not only by heightening access to negative memories, but also by shaping how those memories are interpreted and generalized.

### 6.4 Self-referential processing and default mode network alterations

RNT is intrinsically connected to cognition directed toward the self, which makes changes in self-referential processing especially pertinent. Childhood trauma may shape the neural systems that support self-evaluation, internal mentation, and the interpretation of emotion. Structural neuroimaging studies have found a reduced medial prefrontal cortex volume in individuals who report childhood emotional maltreatment, pointing to potential long-lasting effects on brain regions involved in emotional appraisal and regulation (27). In addition, network-level analyses suggest that childhood maltreatment is linked to altered organization of regions involved in self-processing, including the precuneus, anterior cingulate cortex, and insula (63). These alterations could heighten dependence on internally focused cognitive processing while diminishing the effectiveness of regulatory networks. Conceivably, this configuration could promote sustained self-focused RNT, a central feature of the construct. Nonetheless, direct evidence connecting childhood trauma–related network changes specifically to RNT is still scarce. Accordingly, present neuroimaging findings should be viewed as offering mechanistic plausibility rather than conclusive evidence of causation.

### 6.5 Integrative neurocognitive model: from childhood trauma to RNT and depression vulnerability

Taken as a whole, the available evidence indicates a multidimensional model in which childhood trauma affects multiple neurocognitive systems that operate in interaction. Heightened threat sensitivity could increase exposure to negative cognitive signals, whereas diminished cognitive control may reduce the capacity to disengage from these signals. Concurrently, biases in autobiographical memory and changes in self-referential processing may influence how these experiences are interpreted, thereby strengthening negative self-related narratives. Within this account, RNT may serve as an intermediate cognitive phenotype that connects childhood adversity to vulnerability to depression, rather than functioning as a direct psychological effect of trauma. This view aligns with findings that trauma-associated neurocognitive alterations overlap with mechanisms implicated in depression, including negative cognitive bias, impaired regulation, and excessive self-focused processing (64, 65). Importantly, this model does not imply that trauma exposure inevitably results in RNT or depression. Instead, neurocognitive adaptations may interact with protective factors, environmental context, and individual differences to determine whether RNT becomes persistent and maladaptive.

### 6.6 Sources of heterogeneity and translational implications

Although the evidence base is expanding, multiple factors constrain the ability to generalize findings across studies. Features of trauma such as its type, severity, chronicity, and developmental timing, can yield different neurocognitive outcomes. For instance, childhood trauma-related amygdala responses differ depending on the subtype of abuse and individuals’ clinical status, indicating that a trauma history does not correspond to a single, uniform biological exposure (64). Likewise, heightened neural responses to threatening stimuli in children exposed to family violence may represent adaptive threat detection rather than pathology per se (65). Accordingly, future research should extend beyond the identification of isolated biomarkers and instead investigate dynamic interactions among neural systems, cognitive processes, and environmental conditions. Longitudinal studies integrating neuroimaging, ecological momentary assessment, and computational methods may help determine when trauma-related neurocognitive alterations become associated with persistent RNT. From a clinical standpoint, clarifying these mechanisms could help inform more targeted interventions. Approaches designed to enhance attentional flexibility, bolster executive control, increase the specificity of autobiographical memory, and alter maladaptive self-referential processing may offer promising complementary options. Nonetheless, these potential applications must be empirically supported further before neurocognitive results can be used to develop personalized treatment strategies (**Figure 1**).

**Figure 1:**
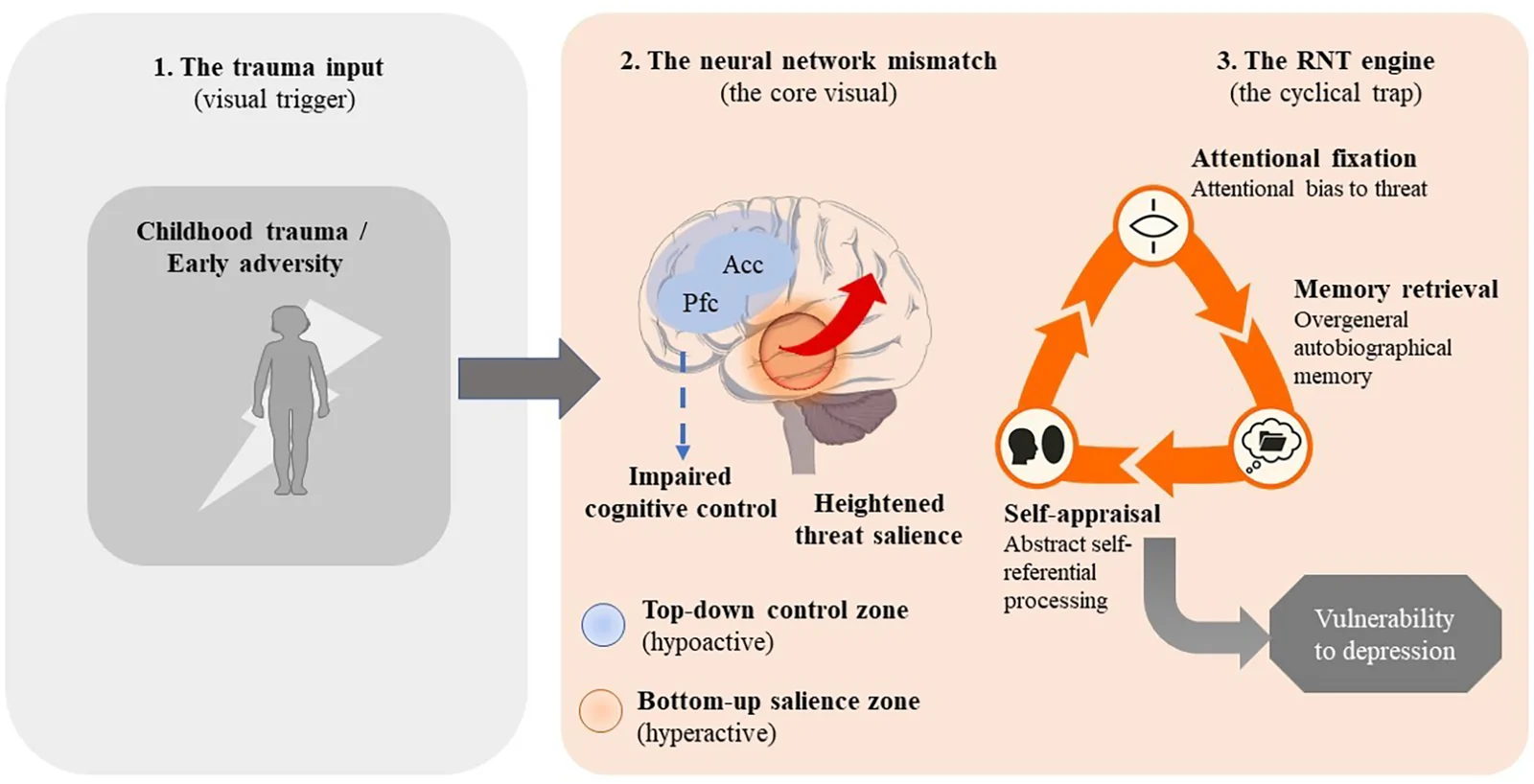
The neurocognitive imbalance model of repetitive negative thinking (RNT). This figure illustrates how early-life adversity disrupts the developmental balance between bottom-up emotional processing and top-down cognitive control networks. Childhood trauma shifts the brain into a state of heightened threat salience characterized by hyperactive limbic processing. Concurrently, structural and functional alterations in prefrontal systems weaken top-down regulatory control, leaving the individual with reduced mental flexibility. This neurocognitive mismatch traps the individual in a self-sustaining RNT loop. The loop is continuously reinforced by threat-related attentional biases, overgeneral autobiographical memory retrieval, and abstract self-referential processing. Over time, this chronic cognitive vulnerability lowers resilience and drives the onset and maintenance of depressive symptoms.

## 7 Stress sensitization and biological embedding of early adversity

Childhood trauma can become biologically incorporated via enduring changes in stress-regulatory systems that shape how individuals cope with later challenges. Rather than indicating discrete abnormalities, these changes embody coordinated adaptations spanning neuroendocrine control, immune signaling, autonomic regulation, neuroplasticity, and large-scale brain networks. Within the stress sensitization framework, early adversity may lower the activation threshold for stress responses, thereby heightening the risk of exaggerated or sustained reactions to subsequent stressors. Crucially, these biological adaptations are not deterministic; they constitute vulnerability routes whose clinical manifestation depends on the timing of development, genetic predisposition, social circumstances, and protective experiences.

### 7.1 HPA-axis dysregulation

The HPA axis is among the main biological systems through which early adversity may shape long-term stress responsiveness. Childhood trauma may change glucocorticoid regulation by influencing feedback sensitivity, receptor function, and the coordination between central and peripheral stress systems. Nevertheless, findings suggest that alterations in the HPA axis are heterogeneous, with effects that can manifest as either heightened reactivity or diminished responsiveness depending on features of the trauma and an individual’s psychiatric status. Early research showed that people exposed to childhood abuse may exhibit stronger adrenocorticotropic hormone and cortisol responses to psychosocial stress, implying enduring sensitization of stress-response pathways (19). In contrast, other studies have found reduced cortisol responses in trauma-exposed individuals who do not have current psychiatric disorders, consistent with possible adaptive downregulation after extended stress exposure (21). Together, these conflicting results indicate that changes in the HPA axis may reflect shifting phases of adaptation rather than a single, uniform biological pattern. Epigenetic processes might help explain the long-lasting persistence of these changes. Experiences of childhood adversity have been linked to alterations in DNA methylation in stress-related genes, including the glucocorticoid receptor gene (NR3C1), which could affect feedback control within the HPA axis (67). Moreover, longitudinal findings indicate that early adversity can combine with later-life exposure to stress to determine cortisol trajectories throughout development (68). Taken together, dysregulation of the HPA axis may constitute one biological route through which childhood trauma is associated with greater susceptibility to depression-related processes. However, changes in cortisol regulation should be considered with caution, as they are shaped by age, sex, medication, sleep, health behaviors, and present environmental stressors.

### 7.2 Chronic inflammation

Chronic, low-grade inflammation has been proposed as one mechanism by which adversity in childhood may become biologically embedded, thereby increasing later susceptibility to depression. Early-life stress may shape immune development by repeatedly engaging inflammatory pathways during critical windows of development, leading to enduring changes in cytokine regulation and immune responsiveness. Nevertheless, inflammatory alterations linked to childhood trauma are not consistent and seem to vary according to factors such as trauma severity, socioeconomic circumstances, health behaviors, and co-occurring psychological symptoms. Large epidemiological studies have found that childhood adversity is associated with higher levels of inflammatory markers in adulthood. Danese et al. reported that people exposed to childhood maltreatment showed greater inflammation, which implies that early adversity may promote lasting immune dysregulation via cumulative biological stress processes (69). Similarly, Slopen and colleagues reported that childhood adversity is linked to higher levels of inflammatory markers, although the strength of these associations differed by the type of adverse experience and by the degree to which such experiences accumulated (70). Inflammatory signaling may also work together with neural systems that are involved in regulating mood. Ongoing activation of cytokines such as interleukin-6 and tumor necrosis factor-alpha can affect neurotransmitter metabolism, HPA activity, and neuroplasticity—processes that have been associated with heightened vulnerability to depression (71). Even so, inflammation should not be viewed as a straightforward explanation for trauma-related depression, because immune changes are likely to be only one element within a wider biopsychosocial framework. More recent evidence also indicates that the immune effects of childhood adversity may be conditioned by resilience factors, including social support and health-promoting lifestyle behaviors. Accordingly, future research should treat inflammation as a changing process that interacts with environmental and psychological influences, rather than as an unvarying biological outcome of exposure to trauma.

### 7.3 Autonomic imbalance

The autonomic nervous system serves as a vital link between exposure to environmental stress and physiological adaptation. Childhood trauma can modify autonomic regulation by disrupting the balance between sympathetic activation and parasympathetic recovery, which may yield reduced responsiveness when confronting emotional or environmental demands. Rather than supporting straightforward hyperarousal frameworks, the current body of evidence indicates that early adversity may compromise the dynamic control of autonomic responses, resulting in either heightened activation or inadequate physiological recovery depending on the situation. Heart rate variability (HRV), a commonly used index of parasympathetic cardiac regulation, has been proposed as a measure of autonomic flexibility and the ability to regulate adaptively. People who have experienced childhood adversity show lower HRV, implying reduced regulatory capacity across physiological systems (72). Likewise, altered autonomic functioning has been reported in individuals with a history of early stress, with results pointing to impaired coordination between sympathetic responding and parasympathetic recovery processes (73). From the standpoint of stress sensitization, reduced autonomic flexibility may help explain why stressful experiences can lead to sustained biological activation. This extended activation may interact with psychological vulnerability factors by keeping physiological arousal elevated, which can make emotional recovery more difficult. Nevertheless, autonomic changes are not specific to childhood trauma and can also arise from the effects of present stress exposure, physical health, sleep disturbance, medication use, and lifestyle factors. The neurovisceral integration model argues that successful emotional regulation relies on communication between prefrontal regulatory systems and autonomic pathways, especially via vagal-mediated mechanisms (74). In line with this, childhood adversity may contribute to later emotional vulnerability in part by impairing physiological adaptability, rather than by causing a single abnormal stress response. Findings from developmental research also indicate that supportive environments and adaptive experiences may shape autonomic pathways, underscoring the plasticity of stress-regulation systems (75).

### 7.4 Neuroplasticity impairment

Early adversity may affect susceptibility to depression by modifying neuroplastic mechanisms that underlie emotional adaptation, learning, and recovery after stress. Neuroplasticity is not confined to structural changes in the brain; it also reflects the ability of neural circuits to adjust their activity to meet environmental demands. Childhood trauma may hinder these adaptive processes by disrupting neurotrophic signaling, impairing synaptic remodeling, and disturbing the balance between neuronal excitation and regulation. That said, these influences are likely to vary with context and to be shaped by developmental stage, the features of the trauma, and later environmental experiences. Brain-derived neurotrophic factor, a central regulator of synaptic plasticity and neuronal survival, has been proposed as one biological route through which early adversity may be linked to altered emotional functioning. Studies in both animals and humans indicate that chronic stress exposure can diminish neurotrophic support, which may in turn restrict adaptive responses in brain regions involved in mood regulation (76). In parallel, early-life stress has been linked to modified expression of genes implicated in neuronal development and plasticity, suggesting that adversity could shape long-term regulatory processes that extend beyond immediate stress responses (77). Structural and functional effects of compromised plasticity may affect brain areas essential for emotion regulation, including the hippocampus and prefrontal cortex. Diminished hippocampal integrity and altered prefrontal regulatory capacity have repeatedly been connected with prolonged exposure to stress; however, results differ substantially across studies and populations (78). Notably, changes related to neuroplasticity should not be construed as irreversible harm, because intervention research indicates that enriched environments, psychological therapies, physical activity, and social support may facilitate beneficial neural adaptations. Taken together, impaired neuroplasticity may constitute a vulnerability pathway through which childhood trauma diminishes resilience to subsequent stressors. Rather than directly causing depression, alterations in adaptive plastic functioning may heighten risk when accompanied by additional biological and psychosocial factors.

### 7.5 Brain network alterations

Beyond regional abnormalities, childhood adversity can affect how large-scale neural networks are organized to support emotional regulation, self-referential processing, and adaptive responses to environmental demands. Current models indicate that susceptibility related to trauma arises not only from alterations in individual brain regions, but also from changed communication among distributed networks, including the DMN, salience network (SN), and central executive network (CEN). Such changes may help explain problems switching among internal reflection, threat detection, and goal-directed regulation. Neuroimaging studies show that childhood adversity is linked to enduring alterations in connectivity patterns involving regions that subserve emotion regulation and stress appraisal. Following early adversity, altered relationships between the amygdala and prefrontal regulatory areas have been reported, indicating diminished top-down control over emotional responses (79). Similarly, exposure to trauma has been associated with atypical DMN organization, especially in areas implicated in self-referential processing and autobiographical interpretation, though the direction and clinical relevance of these results differ among studies (80). Network-level frameworks may help explain how childhood trauma can raise vulnerability to depression without yielding a single, consistent clinical presentation. For instance, heightened SN reactivity could improve detection of emotionally threatening cues, while diminished CEN efficiency may impair adaptive control during periods of stress. Nonetheless, these findings should not be taken as specific biomarkers for trauma-related depression, since comparable network disruptions have been reported across multiple psychiatric disorders. More recent work employing connectomics methods indicates that early adversity may alter developmental pathways of brain network maturation, which in turn could influence the ability to switch flexibly between cognitive and emotional states (64). Nonetheless, longitudinal studies remain necessary to clarify whether changes in neural networks reflect pre-existing vulnerability, the effects of exposure to trauma, or adaptive responses that evolve alongside ongoing environmental demands. Taken together, alterations in brain networks may serve as an integrative pathway linking biological stress sensitization with psychological mechanisms that are relevant to susceptibility to depression. Investigating these changes could support future dimensional frameworks in psychiatry, while also acknowledging marked individual differences in neural responses to childhood adversity (**Figure 2**).

**Figure 2:**
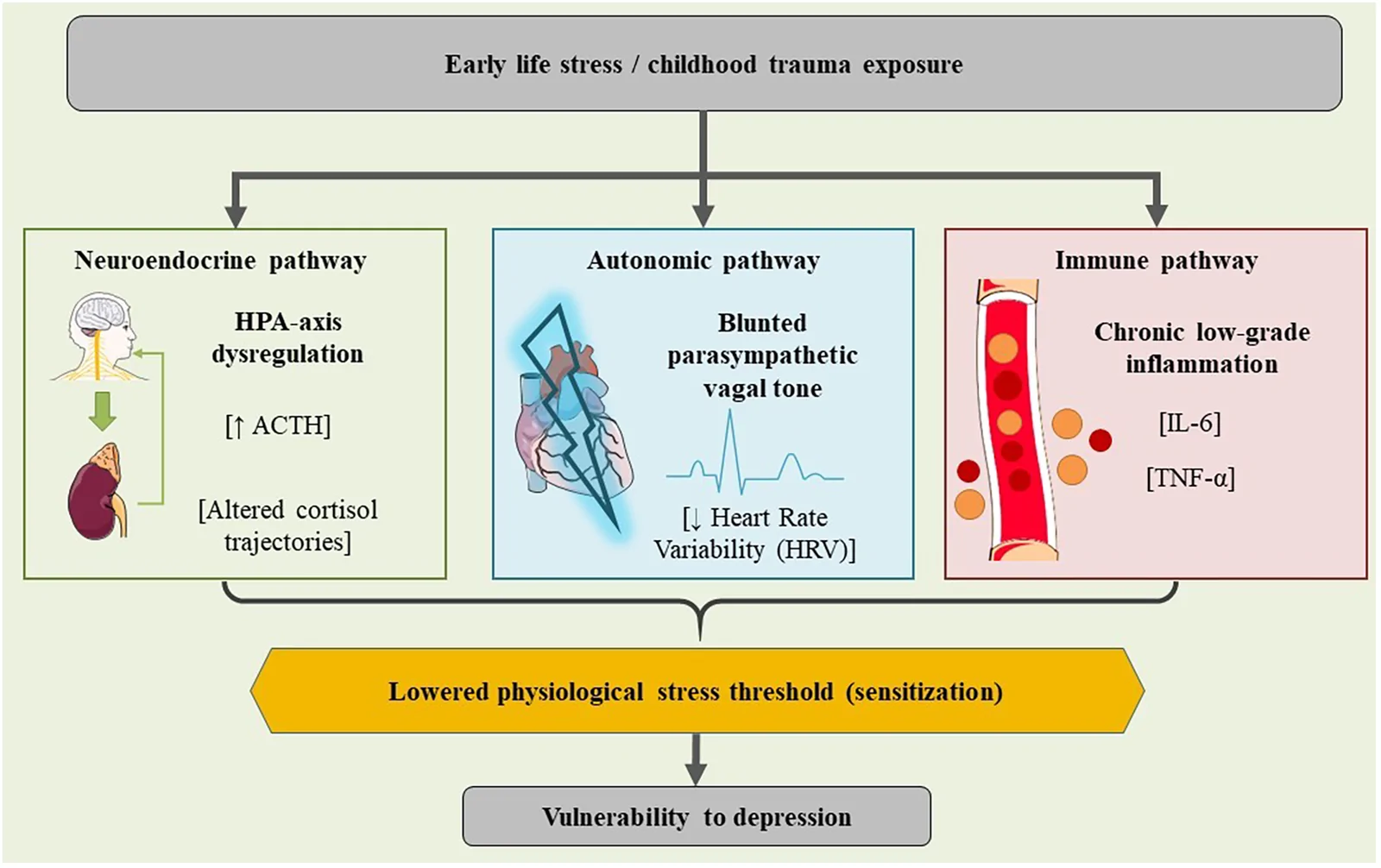
Multi-system biological embedding pathway of early adversity. This schematic depicts the systemic pathways through which childhood trauma becomes biologically incorporated, leading to subsequent stress sensitization. Early life stress acts as a developmental catalyst that simultaneously alters three parallel physiological systems: (Left) neuroendocrine dysregulation within the HPA axis, marked by disrupted cortisol and adrenocorticotropic hormone (ACTH) dynamics; (Center) autonomic imbalance characterized by blunted parasympathetic vagal tone and reduced heart rate variability (HRV); and (Right) a chronic, low-grade inflammatory state indicated by elevated pro-inflammatory cytokines such as interleukin-6 (IL-6) and tumor necrosis factor-alpha (TNF-α). These altered pathways converge to lower the individual’s physiological stress threshold. Ultimately, this heightened biological vulnerability interacts with later-life challenges to increase the risk for depression.

## 8 Integrating the evidence: a transdiagnostic conceptual framework

Depression related to childhood trauma is now increasingly viewed as a multifactorial outcome arising from interactions among developmental experiences, biological adaptations, cognitive processes, and environmental contexts. Rather than reflecting a single, direct, and uniform route from adversity to psychiatric illness, childhood trauma may heighten vulnerability through partially overlapping mechanisms that shape stress reactivity, emotional regulation, and cognitive performance. This account aligns with transdiagnostic models of psychopathology, which highlight shared mechanisms across diagnostic categories rather than relying solely on disorder-specific explanations (4). A key implication of this framework is that early adversity can redirect developmental trajectories by changing how individuals perceive, interpret, and respond to emotional difficulties. McLaughlin and colleagues suggested that childhood experiences marked by threat and deprivation may give rise to distinct developmental pathways, including increased sensitivity to potential threats and fewer opportunities for adaptive learning (4). These changes may not necessarily result in depression, but they could heighten vulnerability when they co-occur with later stressors or reduced protective resources. Within this framework, RNT may serve as an important cognitive link connecting trauma-related vulnerability to depressive symptoms. RNT refers to persistent, hard-to-control negative thought patterns that include rumination and related perseverative cognitive processes. Although it has traditionally been framed as a feature of depression, findings indicate that RNT reflects a transdiagnostic process that operates across multiple forms of psychopathology by sustaining negative emotional states and disrupting adaptive problem solving (39). Accordingly, RNT may act as a mechanism by which earlier biological and emotional vulnerabilities are converted into enduring psychological distress. Emotion regulation processes offer another key integrative element. People who experience adversity during childhood may struggle to choose regulatory strategies that match situational demands, which can lead them to depend more heavily on maladaptive responses such as RNT. Meta-analytic findings show that abnormalities in emotion regulation are broadly linked with psychiatric symptoms, indicating that regulatory inflexibility may function as a shared vulnerability factor rather than a mechanism specific to any single disorder (81). Importantly, these pathways should not be understood as linear or deterministic. Exposure to childhood trauma is neither necessary nor sufficient for the development of depression, and considerable heterogeneity characterizes psychological outcomes after adversity. The Research Domain Criteria framework emphasizes that psychiatric symptoms can arise from interactions among dimensional biological and behavioral systems, rather than from discrete diagnostic categories (7). Consequently, individuals with similar trauma histories may develop depression via different combinations of stress sensitivity, cognitive vulnerability, and regulatory difficulties. From a translational standpoint, this conceptual framework indicates that therapeutic approaches aimed at common underlying mechanisms may work alongside conventional symptom-focused interventions. Interventions designed to lessen RNT, improve the flexibility of emotion regulation, strengthen cognitive control, and bolster adaptive responses to stress may be relevant across trauma-related depressive presentations. Nonetheless, additional longitudinal research is needed to clarify whether these mechanisms function as causal routes, arise as outcomes of long-standing symptoms, or reflect evolving dynamic interactions across development. Overall, childhood trauma can be understood as a developmental context that shapes vulnerability rather than as a single cause of depression. By integrating biological embedding, cognitive processes, and regulatory mechanisms, the framework offers a more comprehensive account of why some individuals exposed to early adversity develop enduring depressive symptoms, while others show resilience.

## 9 Clinical implications

### 9.1 Precision psychiatry and mechanism-informed stratification

Childhood trauma is not a single, consistent risk factor for depression, and growing evidence indicates that clinical outcomes are shaped by the interplay among trauma features, individual vulnerability, and adaptive capacity. Consequently, a precision psychiatry approach may offer greater insight than relying on trauma-based categorization alone. People with comparable trauma histories can nevertheless follow different depressive trajectories, depending on differences in RNT, emotion regulation capacity, threat sensitivity, and cognitive control processes. For instance, some individuals may mainly develop self-focused rumination, while others may display avoidance-oriented cognitive patterns or show impaired emotional recovery. Neurobiological evidence suggests that changes in prefrontal-limbic circuitry, stress-response systems, and memory-related mechanisms may underlie this variability, even though causal links have not yet been fully clarified (26, 82) (**Table 3****).** From a translational standpoint, evaluating dimensions of RNT rather than focusing solely on the presence of childhood trauma may enhance the identification of clinically meaningful mechanisms. Assessments of rumination, cognitive flexibility, emotional regulation, and attentional bias could potentially augment conventional diagnostic methods. However, the current body of evidence is still not adequate to establish whether specific trauma-related cognitive profiles consistently forecast treatment outcomes, underscoring the need for longitudinal research that integrates behavioral, neurobiological, and clinical markers.

**Table 3:** Potential transdiagnostic interventions targeting repetitive negative thinking and related vulnerability mechanisms following childhood trauma.

Intervention	Target population	Study design	Primary therapeutic target	Main findings	Potential relevance to trauma-related RNT	Study
Mindfulness-based cognitive therapy (MBCT)	Individuals with recurrent major depression (n=145)	Randomized controlled trial	Cognitive decentering and reduction of depressive cognitive reactivity	Reduced relapse/recurrence risk, particularly among participants with multiple previous depressive episodes	May reduce trauma-related RNT by promoting disengagement from persistent negative self-focused thinking	(83)
Mindfulness meditation	Distressed adults (n=83)	Randomized controlled trial	Attentional regulation and reduction of rumination	Decreased ruminative thoughts and distress; rumination reduction partially mediated clinical improvements	Supports mindfulness-based approaches for interrupting repetitive negative cognitive cycles	(84)
Concreteness training	Individuals with dysphoria (n=60)	Randomized controlled trial	Abstract and overgeneral negative processing	Increased concrete thinking and reduced depressive symptoms and rumination compared with controls	May counter trauma-associated abstract processing patterns that maintain RNT	(85)
Rumination-focused cognitive behavioral therapy (RFCBT)	Individuals with residual depression (n=42)	Randomized controlled trial	Direct modification of depressive rumination	Improved residual depressive symptoms, with treatment effects mediated by reduced rumination	Provides direct evidence that RNT can serve as a modifiable maintaining mechanism	(86)
Trauma-focused cognitive behavioral therapy (TF-CBT)	Sexually abused children (n=82)	Randomized controlled trial with follow-up	Trauma processing and modification of trauma-related beliefs	Produced greater improvements in depression, anxiety, dissociation, and trauma-related symptoms compared with supportive therapy	May reduce persistent trauma-related cognitive patterns that contribute to RNT vulnerability	(87)
Trauma-focused CBT with trauma narrative components	Children with sexual abuse-related symptoms (n=210)	Randomized controlled trial	Integration of traumatic memories and emotional processing	Improved multiple clinical outcomes across treatment conditions; trauma narrative provided additional benefits for some trauma-related symptoms	Suggests that adaptive processing of traumatic memories may reduce maladaptive repetitive thinking	(82)
Emotion regulation skills training + CBT	Inpatients with major depressive disorder (n=432)	Randomized controlled trial	Emotion regulation capacity and negative affect modulation	Enhanced reductions in depression and improved emotion regulation skills compared with standard CBT alone	May decrease dependence on RNT by improving flexible emotional responses	(88)
Acceptance and commitment therapy (ACT)	Adults with anxiety and depressive symptoms (n=101)	Randomized controlled trial	Cognitive defusion, acceptance, and psychological flexibility	Produced comparable improvements to cognitive therapy, with outcomes associated with increased acceptance and reduced experiential avoidance	Psychological flexibility may weaken the impact of trauma-related repetitive negative thoughts	(89)

ACT, acceptance and commitment therapy; CBT, cognitive behavioral therapy; MBCT, mindfulness-based cognitive therapy; RCT, randomized controlled trial; RNT, repetitive negative thinking; RFCBT, rumination-focused cognitive behavioral therapy; TF-CBT, trauma-focused cognitive behavioral therapy.

### 9.2 Transdiagnostic interventions targeting RNT

A key implication of the transdiagnostic perspective is that RNT may function as a common therapeutic target across depressive and trauma-related disorders, rather than as a symptom limited to any single diagnosis. Multiple psychological interventions seem to affect overlapping mechanisms that help sustain RNT, such as cognitive fusion, abstract processing, attentional rigidity, and deficits in emotional regulation. Mindfulness-based cognitive therapy has been found to lower susceptibility to depressive relapse by strengthening metacognitive awareness and decreasing identification with negative thoughts, especially among people with recurrent depression (83). Likewise, mindfulness-based approaches have shown reductions in rumination and distress, indicating that altering one’s relationship with negative thoughts may be clinically important (84). However, intervention effects should not be taken as evidence that RNT is the only causal route connecting trauma and depression. For instance, rumination-focused cognitive behavioral therapy directly targets repetitive depressive thinking and has demonstrated efficacy in residual depression, yet treatment effects may also reflect wider behavioral and emotional changes (86). Similarly, trauma-focused cognitive behavioral therapy mainly addresses trauma memories and maladaptive beliefs rather than RNT specifically, although gains in trauma-related symptoms may indirectly lessen repetitive negative processing (82, 87). Future research should determine whether integrating trauma-focused strategies with RNT-specific interventions yields added benefit for individuals whose childhood trauma is associated with depression.

### 9.3 Prevention and early identification

The developmental character of childhood trauma-related depression underscores the need for prevention efforts before enduring cognitive patterns take hold. Adverse experiences in childhood can shape early emotional regulation, the allocation of attention, and autobiographical memory processes, thereby increasing susceptibility to later, RNT (64, 87). Accordingly, identifying children and adolescents who show persistent negative, self-focused cognitions, cognitive rigidity, or trouble disengaging from threatening information early on may enable more tailored interventions. However, screening solely for trauma exposure is likely to have modest predictive value, since many individuals with such exposure never develop depression. Preventive strategies should therefore incorporate resilience-related factors, including social support, adaptive coping strategies, and ERF. Interventions delivered through schools and communities that aim to enhance cognitive flexibility and emotional competencies may be particularly promising, even though the evidence on their long-term effects in preventing depression is still emerging.

### 9.4 Digital mental health approaches

Digital interventions can offer scalable ways to target mechanisms related to RNT, especially for people who face obstacles in obtaining psychological treatment. Internet-based cognitive behavioral therapy, mindfulness-based programs, and digital emotion regulation training have shown promise for reducing depressive symptoms and changing maladaptive cognitive processes. Still, it is not yet clear to what degree these approaches specifically influence trauma-related RNT. Digital platforms may be especially useful when paired with adaptive monitoring methods, such as ecological momentary assessment, which can help track changes in rumination, emotional reactions, and contextual triggers. These methods could support more tailored intervention delivery by determining when, and under what conditions, RNT is most likely to remain persistent. That said, bringing such approaches into clinical practice calls for careful evaluation of user engagement, accessibility, privacy, and effectiveness across a wide range of populations.

### 9.5 Future treatment targets and research priorities

Future therapeutic development should progress from merely reducing symptoms to targeting the mechanisms that sustain vulnerability after childhood trauma. Promising targets include fostering cognitive flexibility, supporting the recovery of emotion regulation, altering threat-related attentional biases, and enhancing adaptive autobiographical memory processing. Interventions that combine cognitive training, mindfulness-based strategies, emotion regulation methods, and trauma-focused approaches may offer more comprehensive mechanistic coverage than treatments that address only a single component. Nevertheless, several questions are still unresolved. It remains unclear whether decreasing RNT results in durable prevention of depressive episodes or whether it primarily indicates symptomatic improvement. In addition, variability in trauma type, developmental timing, genetic susceptibility, and environmental context may shape treatment response. Future research employing longitudinal designs and multimodal approaches that integrate behavioral assessments, neuroimaging, and biological markers will be critical for identifying which mechanisms are most clinically actionable.

## 10 Current challenges and future directions

Despite significant progress in elucidating how childhood trauma relates to depression, several key obstacles still constrain the creation of an all-encompassing transdiagnostic framework. Much of the current evidence comes from cross-sectional studies, which makes it challenging to establish the temporal sequence and causal links among childhood trauma, RNT, ERF, neurocognitive dysfunction, and depressive symptoms. Longitudinal research across pivotal developmental windows is therefore needed to determine whether these processes function as antecedents, mediators, or consequences of depression. A further difficulty lies in the pronounced heterogeneity of childhood trauma, including variation in trauma subtype, severity, chronicity, developmental timing, and cumulative exposure; these differences may help explain variability in psychological and neurobiological outcomes. In addition, methodological inconsistencies in assessing trauma, RNT, emotion regulation, and depression make it harder to compare findings across studies and may, at least in part, account for reports that do not align. Future research should also take sociocultural factors into account, since most of the available evidence is derived from Western populations and may not adequately reflect cultural differences in trauma experiences, cognitive processing, emotional regulation, and help-seeking behaviors. The restriction to English-language publications represents a potential source of selection bias, as potentially relevant studies published in other languages may have been excluded, thereby limiting the comprehensiveness and generalizability of the evidence base. Novel approaches including multi-omics technologies, multimodal neuroimaging, and ecological momentary assessment, could yield a more comprehensive understanding of the dynamic mechanisms that link early adversity to depression, although their clinical usefulness still needs further validation. Likewise, artificial intelligence and machine-learning methods may help uncover complex patterns across biological, cognitive, and behavioral data; however, their use is still in an early phase and relies on large, well-characterized longitudinal datasets. Ultimately, combining developmental, neurobiological, and psychological evidence may support the refinement of precision psychiatry approaches. Even so, the present evidence is not sufficient to substantiate definitive mechanistic models, highlighting the need for rigorous prospective studies, standardized assessment protocols, and multidisciplinary research to clarify how transdiagnostic mechanisms can be translated into more effective prevention and intervention strategies.

## 11 Conclusions

Childhood trauma is a significant developmental risk factor for depression; however, the routes by which early adversity increases susceptibility to depressive outcomes are complex, multi-component, and unlikely to be attributable to any single mechanism. In this narrative review, we emphasize RNT as a potentially consequential transdiagnostic process that could link childhood trauma to depression through interplay among ERF, stress sensitization, and neurocognitive changes. Current findings indicate that people who experience childhood adversity may be more prone to persistent maladaptive cognitive patterns, challenges in modifying emotional responses, and increased stress sensitivity—each of which has been linked to greater depressive symptom severity. Despite this, the existing literature is heterogeneous, and the present results should be interpreted with caution due to constraints in study design, measurement methods, and the predominance of cross-sectional evidence. The central conceptual contribution of this review lies in integrating cognitive, emotional, and neurobiological perspectives into a single framework that treats RNT as a possible bridging mechanism rather than as a standalone symptom or as a direct consequence of trauma exposure. This viewpoint may facilitate progress beyond conventional disorder-specific models by highlighting shared processes that underlie depressive vulnerability in people with different trauma histories. Clinically, recognizing RNT and associated regulatory difficulties may offer valuable targets for assessment and intervention; however, additional research is necessary to establish whether altering these mechanisms yields meaningful and sustained improvements in depression outcomes. Future research should emphasize longitudinal and mechanistic studies designed to clarify causal pathways linking childhood trauma, RNT, regulatory processes, and depressive symptoms. Incorporating neuroimaging, multi-omics approaches, ecological momentary assessment, and computational methods may further sharpen understanding of individual differences in trauma-related depression. Ultimately, a more accurate account of transdiagnostic mechanisms could help inform the creation of individualized prevention and treatment approaches, while also acknowledging that the current evidence does not yet substantiate any conclusive causal models.
